# The Measure of the Family Caregivers’ Experience

**DOI:** 10.3390/ijerph15092040

**Published:** 2018-09-18

**Authors:** Mercedes Guilabert, Paloma Amil, Asunción González-Mestre, Esther Gil-Sánchez, Anna Vila, Joan Carles Contel, Juan Carlos Ansotegui, Olga Solas, Ma Teresa Bacigalupe, Paloma Fernández-Cano, Marisa Arteagoitia, José Joaquín Mira

**Affiliations:** 1 Health Psychology Department, Universidad Miguel Hernández de Elche, Comunidad Valenciana, 03202 Elche, Spain; jose.mira@umh.es; 2 Programa Pacient Expert Catalunya^®^, Programa de Prevenció i Atenció a la Cronicitat Direcció General de Planificació i en Salut, Departament de Salut, Cataluña, 08028 Barcelona, Spain; pamil@gencat.cat (P.A.); assumpcio_gonzalez@gencat.cat (A.G.-M.); egils@gencat.cat (E.G.-S.); jccontel@gencat.cat (J.C.C.); 3 Servei de Valoracions, Sub-Direcció General d’Atenció i de Promoció de l’Autonomia Personal Direcció general de Protecció Social, Departament de Treball, Afers Socials i Famílies, Cataluña, 08019 Barcelona, Spain; avilar@gencat.cat; 4 Osakidetza, Servicio Vasco de Salud, País Vasco, 01006 Vitoria-Gasteiz, Spain; juancarlos.ansoteguiperez@osakidetza.eus (J.C.A.); mariateresa.bacigalupeartacho@osakidetza.eus (M.T.B.); 5 Consultora de Políticas Públicas y Gestión de Organizaciones, Castilla la Mancha, 45112 Toledo, Spain; olgasolasg@gmail.com; 6 Merck Sharp & Dohme (MSD), 28027 Madrid, Spain; paloma_fernandezcano@merck.com; 7 Fundación Vasca de Innovación Sanitaria, País Vasco, 48902 Bilbao, Spain; marteagoitia@bioef.org; 8 Alicante-Sant Joan Health District, Comunidad Valenciana, 03550 Alicante, Spain; 9 REDISSEC, Red de Investigación en Servicios de Salud en Enfermedades Crónicas, Comunidad Valenciana, 46020 Valencia, Spain

**Keywords:** caregivers, chronic disease, quality assurance, health services evaluation

## Abstract

*Objective*: Design and validate a measure of the experience of family caregivers with the integrated care that receive the persons they care for. *Methods*: The new instrument for measuring the experience of caregivers is based on the Instrument to Evaluate the EXperience of PAtients with Chronic Diseases (acronym in Spanish: IEXPAC) scale instrument. With the qualitative technique of the discussion group, nine professionals and eight caregivers assessed the face validity of the instrument and they advised on issues to explore and the measuring scale to use. The instrument’s items were analyzed individually, as well as its consistency, reliability, and construct and empirical validity. *Results*: 235 caregivers responded, of which 186 (79%) were women. The average age of the persons under their care was 83.9 years (SD 9.7). The scale’s score when eliminating its items one by one ranged between 38.6 and 41.1. The factorial saturations of the items ranged between 0.53 and 0.82. Cronbach’s alpha (12 elements) was 0.88 and the Kuder-Richardson coefficient was 0.91. The factorial solution explained 64.3% of the total variance and allowed isolating two factors (with 11 items with saturations greater than 0.65): care for the patient, and care for the caregiver. The internal consistency of both factors was greater than 0.80. The scale’s score was 41.1 (SD 9.7). *Conclusions*: The Caregivers Experience Instrument combines acceptability, ease of comprehension, and perceived usefulness for the caregivers. It has adequate internal consistency, reliability, and construct and empirical validity.

## 1. Introduction

The increasing longevity within the population and the rapid growth in the number of persons who suffer from chronic conditions, together with the phenomena of pluripathology and the associated greater fragility and complexity, constitute an important focus of attention in the reforms of the health and social systems in the modern societies. [1,2,3]. In Spain, it is estimated that there are almost 20 million people with chronic health problems for whose care three-quarters of the country’s health resources are allocated [4].

In the 1990s, new organizational models of caring for chronicity and interventions began being proposed to provide an appropriate and proportional response to this phenomenon [5,6]. Attention was placed on the wellbeing of the person and in promoting his or her active participation to maintain that wellbeing within the framework of integrated care [7]. With this purpose in mind, the Instrument for the Assessment of Chronic Care Models (IEMAC-ARCHO) was designed in Spain in 2012.

IEMAC [8] is an instrument for evaluating the new ways of organizing the response of health and social systems to the phenomenon of chronicity, designed and validated within the Spanish context. This instrument allowed the identification of gaps in our organizational model and in the interventions to address the needs of the most fragile and chronic persons and those of the persons who live with them [9]. One of these gaps is the care for persons who care for others, normally, family members.

Within our surroundings, family caregivers take care of the daily needs of 75% of the persons older than 65 with pluripathology, fragility, and certain complexity, and this figure might be even higher [10]. Their intervention is essential for meeting the needs of these people.

Family caregivers tend to be mostly women (around 84%), with an average age of 53, housekeepers (44%), lack their own income and, in most cases, are daughters or spouses of the person under their care. Around 60% have low levels of education (without any or only primary level studies) [10]. The life of the caregiver, in most cases, revolves around the person in their care, and they quit jobs and live with the patient, fulfilling their role without any type of schedule [11].

The amount of care that a person may require has been related with the repercussions upon the physical, emotional, social, and family health in their caregivers. Therefore, there is a need to personalize this assessment in terms of the experience, capacity and context of the caregiver [12]. The burden that caregivers are subjected to can be related to the presence of endocrine, nutritional, and metabolic conditions, resulting largely from the social isolation, lack of free time, low quality of life, and even a deteriorating economic situation [13,14,15,16,17]. To measure this burden, there are some scales, being the Zarit Scale, the most used [18,19]. These scales measure the consequences of the caring experience, but do not evaluate the specific aspects that can improve their experience or alleviate their physical and emotional burden [20,21].

Family caregivers do not usually have information about support resources, and doubts persist as to whether they receive sufficient attention and support from health and social institutions. This study’s objective was to design and validate a scale to measure the experience for family caregivers with the integrated care received by the persons in their care.

## 2. Method

This study’s protocol was approved by the Ethics Committee of Clinical research at the San Juan de Alicante University General Hospital (18/303 on 5 February 2018). The new instrument for measuring the experience of caregivers is based on the Chronic Patient Experience Evaluation Instrument (IEXPAC) [22]. The design of this new instrument included qualitative techniques to define the scope of this instrument, content validity, legibility, and face validity. The validation included quantitative techniques to determine metric properties of each element, consistency, reliability, and construct and empirical validity analysis.

Family caregivers dedicate a portion of their time to provide care for dependent persons that are unable to perform the Basic Activities of Daily Living (BADL), within their home and in the hospital, if needed. They are family members or persons from within their environment and not linked to a professional care service [23]. Dependency was defined as the permanent status a person finds oneself in, which for reasons due to age, illness, or disability, and linked to a loss of physical, mental, intellectual, or sensory autonomy, requires significant assistance for carrying out the BADL [23]. The Spanish Personal Autonomy and Dependent Care Law (39/2006) recognizes different degrees of dependency, providing some type of support, mainly financial, for the most serious situations.

### 2.1. Face, Legibility, and Content Validity

#### 2.1.1. Professionals’ Views

A multiprofessional panel was formed that consisted of nine care professionals from the regional health services of Andalucía, Catalonia and the Basque Country with the following profiles: physicians, nursing, social workers, case managers, and integrated area managers. The purpose of this validation phase was to evaluate the relevance/appropriateness of the scale items, the ease of understanding its items (friendliness), and the usefulness of this measure of the caregiver’s experience for the organization and professional practice. The contributions were included in the final prototype.

#### 2.1.2. Caregivers’ Views

A qualitative study was carried out (discussion group technique) with participation by a random selection of eight family caregivers. The participants individually assessed the items from the IEXPAC instrument (except for the item on accessibility to the clinical history by the Internet), and two items added to explore whether the professionals who regularly attended to the patient were also concerned about the family caregiver own health and wellbeing and the magnitude of the emotional burden of caring for another person. There were also two possible and alternative response scales (*Always* to *Never* versus *Completely Agree* to *Completely Disagree*). In all, 16 items were assessed in terms of their perceived usefulness for making a measurement that reliably reflected their assessment about whether they receive integrated care and their suitability for measuring their experience as caregivers. Additionally, a group discussion was held about possible unexplored areas. Finally, the degree of comprehension of the scale elements was assessed by asking about possible alternative wording. The comprehension of the elements was assessed on a scale from 1 to 5. The scale’s score was calculated by simply adding the score of each element.

#### 2.1.3. Validation

A random sample of 288 subjects were invited to participate during the field study (sampling error 5%, level of significance of 95%). Subjects were family caregivers of patients receiving care at healthcare centers in Catalonia, Valencian Community and Basque Country (belonging to three regional health services).

#### 2.1.4. Item Analysis

The ceiling and floor effects were analyzed for each item, the magnitude of its correlation with the total score (rejecting values less than 0.50), its impact on the consistency of the scale upon eliminating the item, and its factorial saturation.

#### 2.1.5. Internal Consistency and Reliability

Cronbach’s alpha was calculated for the entire scale and for each of the isolated factors in the factor analysis. As a measure of reliability, the Kuder-Richardson formula (KR20) was used considering odd-even and split-half methods of the items.

#### 2.1.6. Construct Validity

Factor analysis of principal components was carried out by subjecting the resulting matrix to rotation by the varimax procedure. Factorial saturations greater than 0.55 were considered acceptable.

#### 2.1.7. Empirical Validity

The scale’s predictive ability was estimated by analysis of variance (ANOVA), considering the degree of recognized patient dependence as independent variable, and hypothesizing that as this level increased, the burden for the caregiver (related to a negative experience) also increased [24].

#### 2.1.8. Translation-Back Translation

The Caregiver Experience Instrument with 12 items in Spanish and four additional questions related to situations that are common to this population group (hospital admission, going to the emergency room, home care, and care by social services) was translated into English by bilingual individuals with more than 15 years of care experience. This version was translated to English, independently, by a native English speaker with experience in English-Spanish-English translation of health reports and papers. Both versions were compared by two investigators to resolve potential inconsistencies. In the compared versions, two levels were determined: total agreement (where all items from the Spanish version to English and then back to Spanish coincide), partial agreement (where most items and the meaning that is intended with the formulation of the item coincide), or no agreement (the majority of the items do not coincide and the meaning that is intended with the formulation of the item is different).

## 3. Results

### 3.1. Instrument Design

In the group sessions to determine the face and content validity, nine professionals (5 women (55.5%) and eight family caregivers participated (average age, 67 years; 100% women, 16 years’ average experience as caregiver). In this sample, the patients cared for presented the following main health conditions: Alzheimer’s in 62% of the cases, mental illness in 37%, and COPD (Chronic Obstructive Pulmonary Disease) in 25%. The professionals determined the relevance of the questions (88.9%) and use of this scale in clinical practice (100%).

The areas explored by the set of scale items were thought to address relevant aspects of their experience as caregivers. A five-point Likert response scale (range from Never to Always) was assessed as being the simplest and most comprehensible (5, 62%) versus the Completely Agree to Completely Disagree scale (3, 37%). For 87%, the presentation format that would most facilitate being answered was with pencil and paper while 50% thought an Internet format would be best. Of the 16 questions, 10 obtained an average of 5 points in the measure of comprehension. The scores ranged between 4.3 and 5 points. Modifications to wording were proposed for two items to improve their comprehension.

### 3.2. Instrument Validation

In the field study, 235 caregivers responded (response rate of 81.6%), of which 186 (79.1%) were women. The average age of the person in their care was 83.9 years (SD 9.7) and mostly women (141, 60%). The caregivers’ average age was 60.6 years (SD 11.7) and the average time that they had cared for the person was 6.8 years. In most cases, the patient and caregiver lived together (163, 69.4%) (Table 1).

The analysis of the scale items revealed variations of as many as 2.5 points in the scores (Table 2). No items showed a floor or ceiling effect (more extreme values were identified in the “almost never” response option in the case of two items: *They respect the lifestyle of the person I care for* and *They ask me about and help me follow the treatment plan for the person in my care*, with less than 3% choosing this response option) that recommended their exclusion. The average scale score was 42.9 points (SD 10.1). The scale scores when eliminating their items one by one ranged between 38.6 (*They are concerned about the wellbeing of the person in my care*) and 41.1 (*They help me find information over the Internet*) points. Pearson’s correlation of each item with the total scale score was greater than 0.50 with the exception of the *They help me find information over the Internet* item (0.27). The factorial saturations of the items on the resulting scale ranged from 0.53–0.82, except in the case of the *They help me find information over the Internet* item, which was 0.12.

The total value of Cronbach’s alpha (for the 12 items) was 0.88. The scale consistency upon eliminating its items one by one ranged from 0.86–0.89 (Table 3). The value of the KR20 coefficient for the division by halves was 0.91 considering odd-even items (Cronbach’s alpha for even items was 0.79 while for odd items it was 0.75) and 0.75 considering the first 6 items with respect to the set of the second six (Cronbach’s alpha for the first six items was 0.81 while for the next six it was 0.84).

Two factorial solutions were analyzed: the first with 12 items while the second had 11, and excluded *They help me find information over the Internet* due to its low factorial saturation. Both factorial solutions coincided in the extraction of two factors with similar structures and factor loadings. 

The second factorial solution, with all the items with factor loadings greater than 0.60 explained 64.3% of the total variance (Table 4) and permitted isolating two factors: attention for the patient (Factor 1) and attention for the caregiver (Factor 2). The first focused on the attention that the patient receives from the caregiver’s perspective whereas the second focused on the attention that the own caregiver receives. The internal consistency of both factors was greater than 0.80. The score for this version of the scale with 11 items was 41.1 (SD 9.7). Empirical validity values are presented in Table 5.

The normative values of reference in the scale are shown in Table 6, Table 7, Table 8, Table 9, Table 10 and Table 11.

### 3.3. Translation-Back Translation

The IEXPAC instruments was adapted to be used in English. The translation from English to Spanish yielded an acceptable level of agreement in both versions. All items were placed into the terms of partial agreement in their great majority (12) and total agreement (4) (Table 12). Appendix A includes both versions of this instrument.

## 4. Discussion

The Caregivers Experience Instrument is a tool that combines acceptability, ease of comprehension, and perceived utility for caregivers. It has adequate internal consistency, reliability, and construct and empirical validity. The measurements that this instrument offers do not depend upon individual factors of either the patient or the caregiver, but rather upon the type, scope, and perceived results of the health and social interventions that they receive. Thus, it is a solid and valid instrument for periodically analyzing the experience of individuals who assume the role of caregiver in the home of a person who suffers one or more chronic conditions.

The instruments that have been developed so far for learning about the experience of caregivers have explored, fundamentally, the emotional burden that caring for a family member entails [25]. They have identified affective disorders and insomnia as the most frequent consequences that result from continually caring for a family member over time [25], or they have focused on aspects related to activation of the patient but not for the caregiving person [26,27]. The Caregivers Experience Instrument, unlike other instruments, explores elements of integrated health and social care for both the patient and caregiver alike, which can facilitate the caregiving experience and therefore manage their burdens and improve their quality of life.

This measure can contribute to achieve integrated care by incorporating emotional, informational, and personal needs of caregivers. They usually do not receive sufficient attention to enable them to perform their functions alleviating unnecessary burdens. It can be a suitable instrument for research the provision of services, to learn of the situation of coordination of care and support for caregivers. Its reading has the potential to empower caregivers by providing them with key points in monitoring and care for both, the person in their care and themselves.

The introduction of new organizational models in caring for chronicity has taken place alongside the substitution of the traditional paradigm (“all for the patient, but without the patient”) for a different one based on respect for the person more in line with the aphorism of “nothing about me without me” [28,29,30]. The metrics around the concept of experience of the person lead to an evaluation of aspects relevant for the redesign of structures, organizations, and procedures that the IEXPAC scale included in its evaluation scheme. For this reason, its validation and subsequent use in the evaluative frameworks of health and social systems that work in integrated care settings is fundamental. Likewise, these types of measures must be complemented with qualitative strategies and methodologies that reflect the voices of persons and caregivers to construct an adequate narrative of integrated care centered on the person and her caregiver. The results of this study justify that greater attention be paid toward family caregivers with a double objective: to care for their personal needs (affective and concerning knowledge for carrying out their work) and to modify care processes to include their vision of how providing care at health centers and within a patient’s home. 

Limitations. Most of the subjects who responded to the scale were caring for a patient with a neurological disorder, but none were caring for a person with a mental disorder; neurological conditions, such as Alzheimer’s and dementia, represent the majority of patients who need caregivers since their level of autonomy becomes increasingly limited. Not all caregivers were aware of the degree of dependency of their family member, and this limits the size of this subgroup in the comparisons made. The data referring to the patient’s medication or to the amount of time that the caregiver has been carrying out this labor are estimated by the own caregiver.

## Figures and Tables

**Table 1:** Sample description.

	Caregiver		Person They Care for	
	Frequency	Percentage	Frequency	Percentage
Female	186	79.1	141	60
Male	45	19.1	62	26.4
No answer	4	1.8	32	13.6
Total	235	100	235	100
	Average	SD	Average	SD
Age	60.6	11.7	83.9	9.7
Time as caregiver (in months)	82.6	61.9		
Medications taken daily			3.5	1.1
**Do you live with the person you currently care for?**				
	Frequency	Percentage		
No	72	30.6		
Yes	163	69.4		
Total	235	100		
Does the person you care for have a recognized degree of dependency *?				
	Frequency	Percentage		
No	72	30.6		
Yes	163	69.4		
Total	235	100		
**If affirmative, the degree of dependency is**				
	Frequency	Percentage		
Degree 1	22	9.4		
Degree 2	64	27.2		
Degree 3	64	27.2		
Do not know	85	36.2		
Total	235	100		
**Patient’s primary health condition**				
	Frequency	Percentage		
Respiratory	19	8.1		
Oncological	9	3.8		
Neurological	133	56.6		
Nephrological	6	2.6		
Endocrinological	8	3.4		
Cardiovascular	33	14		
Other	18	7.7		
No answer	9	3.8		
Total	235	100		

* Degree of dependency refers to a permanent state in which people need the attention of other people to perform the basic activities of daily life for various reasons (age, illness, disability, etc.).

**Table 2:** The Caregivers Experience Instrument items’ scores.

Items	Average	Standard Error	SD	% = 5 ^+^
1. They respect the lifestyle of the person I care for.	4.1	0.1	1.1	45.5
2. They are coordinated to offer us good care.	4.0	0.1	1.2	43.0
3. They help me find information over the Internet.	1.8	0.1	1.2	6.8
4. I now know how to look after him/her better.	3.9	0.1	1.1	38.7
5. They ask me about and help me follow the treatment plan of the person in my care.	4.3	0.1	1.1	57.9
6. We agree on the most important objectives of his/her care to control his/her health problems better.	4.0	0.1	1.3	45.5
7. They ensure that he/she takes the medication correctly.	4.3	0.1	1.3	68.1
8. They are concerned about the wellbeing of the person in my care.	4.3	0.1	1.1	60.0
9. They are concerned about my health and wellbeing.	3.3	0.1	1.5	31.1
10. They are concerned about my emotional and physical burden.	3.1	0.1	1.5	25.5
11. They inform me about health and social resources that can help me.	3.5	0.1	1.5	34.0
12. They encourage me to talk to other caregivers.	2.4	0.1	1.5	18.7
**Additional questions**				
13. They care about the person in my care upon their arrival home after being in hospital.	2.3	0.1	1.7	20.9
14. I know where I have to contact when the person in my care has an emergency.	3.3	0.1	1.8	45.1
15. They care for the person in my care well in his/her home.	3.7	0.1	1.7	53.2
16. Social services are coordinated with the health services to provide us with good care.	2.3	0.1	1.7	20.0

N = 235. Scale from 1 to 5. ^+^ Percentage of subjects who answered with the *always* response option (score = 5).

**Table 3:** Analysis of the items. Average, item-total correlation, and consistency by eliminating the element.

Items	Scale Average If the Item Is Eliminated	Scale Variance If the Item Is Eliminated	Item-Total Corrected Correlation	Cronbach’s Alpha If the Item Is Eliminated
1. They respect the lifestyle of the person I care for.	38.8	90.9	0.53	0.87
2. They are coordinated to offer us good care.	39.0	89.3	0.54	0.87
3. They help me find information over the Internet.	41.1	94.9	0.27	0.89
4. I now know how to look after him/her better.	39.0	88.4	0.61	0.87
5. They ask me about and help me follow the treatment plan of the person in my care.	38.7	87.3	0.70	0.86
6. We agree on the most important objectives of his/her care to control his/her health problems better.	39.0	86.4	0.64	0.87
7. They ensure that he/she takes the medication correctly.	38.7	87.8	0.57	0.87
8. They are concerned about the wellbeing of the person in my care.	38.6	88.7	0.65	0.87
9. They are concerned about my health and wellbeing.	39.6	81.8	0.70	0.86
10. They are concerned about my emotional and physical burden.	39.8	81.8	0.69	0.86
11. They inform me about health and social resources that can help me.	39.5	85.0	0.58	0.87
12. They encourage me to talk to other caregivers.	40.5	87.3	0.46	0.88

N = 235.

**Table 4:** Construct validity. Factorial analysis of principal components and varimax rotation.

Items/Factors	1	2
5. They ask me about and help me follow the treatment plan of the person in my care.	0.81	
6. We agree on the most important objectives of his/her care to control his/her health problems better.	0.81	
4. I now know how to look after him/her better.	0.77	
1. They respect the lifestyle of the person I care for.	0.74	
2. They are coordinated to offer us good care.	0.72	
8. They are concerned about the wellbeing of the person in my care.	0.70	
7. They ensure that he/she takes the medication correctly.	0.67	
10. They are concerned about my emotional and physical burden.		0.87
11. They inform me about health and social resources that can help me.		0.84
9. They are concerned about my health and wellbeing.		0.83
12. They encourage me to talk to other caregivers.		0.73
Explained variance (total 64.3%)	48.2	16.1
Cronbach’s alpha	0.88	0.86
Extraction method: principal component analysis.		
Rotation method: varimax standardization with Kaiser.		

**Table 5:** Empirical/predictive validity of the Caregivers Experience Instrument.

		Recognized Degree of Dependency			Confidence Interval (95)		*p* =
		N	Average	SD			
Total score	Degree 1	22	43.3	6.9	40.2	46.4	0.003
	Degree 2	64	42.7	9.5	40.3	45.1	
	Degree 3	64	37.1	11.5	34.2	39.9	
	Total	150	40.4	10.5	38.7	42.1	
Factor 1	Degree 1	22	30.0	4.6	27.9	32.0	0.004
	Degree 2	64	29.7	6.1	28.1	31.2	
	Degree 3	64	26.0	7.5	24.2	27.9	
	Total	150	28.2	6.8	27.1	29.2	
Factor 2	Degree 1	22	13.4	3.4	11.9	14.9	0.042
	Degree 2	64	13.0	5.2	11.8	14.3	
	Degree 3	64	11.0	5.3	9.7	12.4	
	Total	150	12.2	5.1	11.4	13.1	

**Table 6:** Caregivers Experience Instrument averages and standard deviations by sex, the patient lives alone or not, recognized degree of dependency.

	Total Score		Factor 1		Factor 2	
Caregiver’s sex	Female	Male	Female	Male	Female	Male
Average	41.5	40.3	29.1	27.7	12.4	12.6
SD	10.0	8.7	6.4	5.4	5.3	4.1
Patient’s sex	Female	Male	Female	Male	Female	Male
Average	41.3	40.9	29.1	27.8	12.1	13.1
SD	9.5	11.0	6.0	7.1	5.2	5.2
Do you live with the person you currently care for?	Yes	No	Yes	No	Yes	No
Average	41.4	40.6	28.6	29.1	12.8	11.4
SD	9.9	9.3	6.4	5.9	5.2	4.8
Does the person you care for have a recognized degree of dependency?	Yes	No	Yes	No	Yes	No
Average	40.3	43.0	28.2	30.1	12.1	13.0
SD	10.3	8.0	6.7	4.9	5.1	4.9

**Table 7:** Caregivers Experience Instrument averages and standard deviations by degree of dependence.

Recognized Degree of Dependency	Total Score			Factor 1			Factor 2		
	Degree 1	Degree 2	Degree 3	Degree 1	Degree 2	Degree 3	Degree 1	Degree 2	Degree 3
Average	43.3	42.7	37.1	30.0	29.7	26.0	13.4	13.0	11.0
SD	6.9	9.5	11.5	4.6	6.1	7.5	3.4	5.2	5.3

**Table 8:** Caregivers Experience Instrument averages and standard deviations by number of medications taken daily.

Number of Medications Taken Daily	Total Score					Factor 1				Factor 2					
	1–2	3–4	5–6	7 or More	None	1–2	3–4	5–6	7 or More	None	1–2	3–4	5–6	7 or More	None
Average	37.5	38.4	41.1	42.3	42.3	25.2	26.7	29.4	29.6	29.4	12.4	11.6	11.7	12.6	12.9
SD	13.5	11.7	8.7	8.4	9.8	8.8	7.3	5.3	5.5	6.2	5.8	6.2	5.4	4.4	5.0

**Table 9:** Caregivers Experience Instrument averages and standard deviations by Primary health condition of the person cared for.

	Total Score								Factor 1								Factor 2				
Primary Condition of Person cared for	Respiratory	Oncological	Neurological	Nephrological	Endocrine	Circulatory	Other	Respiratory	Oncological	Neurological	Nephrological	Endocrine	Circulatory	Other	Respiratory	Oncological	Neurological	Nephrological	Endocrine	Circulatory	Other
Average	43.5	43.4	40.0	46.0	43.6	44.4	40.9	31.3	31.9	28.0	31.0	29.6	30.5	28.7	12.2	11.6	12.0	15.0	14.0	13.9	12.2
SD	7.3	5.8	10.0	7.6	13.7	8.2	10.1	4.0	3.0	6.2	4.4	9.9	4.8	6.6	4.2	5.2	5.4	3.3	4.8	4.5	5.3

**Table 10:** Caregivers Experience Instrument averages and standard deviations. Patients´ and Caregivers´ age.

**Caregiver’s Age**	**Total Score**				**Factor 1**				**Factor 2**			
	**18–40**	**41–60**	**61–70**	**Over 70**	**10–40**	**41–60**	**61–70**	**Over 70**	**10–40**	**41–60**	**61–70**	**Over 70**
Average	41.0	40.4	41.2	43.5	30.5	28.8	28.2	29.5	10.5	11.6	13.1	14.0
SD	8.1	9.6	11.0	8.4	3.3	6.1	7.2	5.6	5.7	5.2	5.0	4.5
**Patient’s age**	**Total score**				**Factor 1**				**Factor 2**			
	**18–40**	**41–60**	**61–70**	**Over 70**	**10–40**	**41–60**	**61–70**	**Over 70**	**10–40**	**41–60**	**61–70**	**Over 70**
Average	44.0	48.5	37.1	41.3	30.0	34.3	25.1	28.9	14.0	14.3	12.0	12.3
SD	4.2	3.0	14.7	9.5	4.2	1.0	10.3	5.9	0.0	2.2	5.1	5.2

**Table 11:** Caregivers Experience Instrument averages and standard deviations. Time as caregiver.

Time as Caregiver	Total Score				Factor 1				Factor 2			
	<2 Years	2–4 Years	>4–7 Years	>7 Years	<2 Years	2–4 Years	>4–7 Years	>7 Years	<2 Years	2–4 Years	>4–7 Years	>7 Years
Average	41.8	42.7	40.1	41.0	29.7	29.4	28.1	28.7	12.1	13.4	12.0	12.4
SD	8.3	11.2	9.0	10.1	6.0	6.6	6.1	6.3	5.3	5.5	4.7	5.1

**Table 12:** Caregivers Experience Instrument translation and back translation.

Scale Items	Original Translation/Back Translation
1. They respect the lifestyle of the person I care for.	Partial agreement
2. They are coordinated to offer us good care.	Partial agreement
3. They help me find information over the Internet.	Partial agreement
4. I now know how to look after him/her better.	Partial agreement
5. They ask me about and help me follow the treatment plan of the person in my care.	Partial agreement
6. We agree on the most important objectives of his/her care to control his/her health problems better.	Partial agreement
7. They ensure sure that he/she takes the medication correctly.	Partial agreement
8. They are concerned about the wellbeing of the person in my care.	Partial agreement
9. They are concerned about my health and wellbeing.	Total agreement
10. They are concerned about my emotional and physical burden.	Total agreement
11. They inform me about health and social resources that can help me.	Partial agreement
12. They encourage me to talk to other caregivers.	Total agreement
Additional questions	
13. They care about the person in my care upon their arrival home after being in hospital.	Partial agreement
14. I know where I have to contact when the person in my care has an emergency.	Total agreement
15. They care for the person in my care well in his/her home.	Partial agreement
16. Social services are coordinated with the health services to provide us with good care.	Partial agreement

## Appendix A

### Appendix A.1. English Translation of the Questionnaire

**EXPERIENCE OF THE FAMILY CAREGIVER OF PEOPLE WITH CHRONIC DISEASES OR DEPENDENCE**

In the questions below, describe how often you experience these situations (mark the appropriate box with an X). It is important to focus your answers on a specific person with whom you have had significant experience as a caregiver **in the last 6 months**.

	**Never**	**Almost Never**	**Sometimes**	**Almost Always**	**Always**
**1. They respect the lifestyle of the person I care for** The healthcare professionals who care for the person in my care ask me about his/her needs, habits, and preferences to adapt his/her treatment and care plan.	□	□	□	□	□
**2. They are coordinated to offer us good care** The healthcare professionals who care for the person in my care at the health center and those who care for him/her at the hospital talk to each other and coordinate to improve his/her wellbeing and quality of life and those of the family.	□	□	□	□	□
**3. They help me become informed over the Internet** The healthcare professionals who care for the person in my care inform me about websites and Internet forums that I can trust to better understand his/her disease, its treatment, and the consequences it may have on his/her life.	□	□	□	□	□
**4. I now know how to look after him/her better** With the support of the healthcare and social professionals caring for the person in my care, I feel I have more confidence in my ability to take care of him/her, manage his/her health problems, and approach his/her situation better.	□	□	□	□	□
**5. They ask me about and help me follow the treatment plan of the person in my care** I review with the healthcare professionals who care for the person in my care the adherence to his/her treatment and care plan, and if I have questions, they answer them.	□	□	□	□	□
**6. We agree on the most important objectives of his/her care to control his/her health problems better** I have been able to discuss and agree with the healthcare professionals who care for the person in my care the most important health and social problems and how to manage them adequately to maintain his/her quality of life.	□	□	□	□	□
**7. They ensure that he/she takes medications correctly** The healthcare professionals caring for the person in my care review with me how to administer medications and review with me if he/she is taking them correctly and how he/she is feeling.	□	□	□	□	□
**8. They are concerned about the wellbeing of the person in my care** The healthcare and social care professionals who care for the person in my care are concerned about his/her quality of life and I feel they are committed to improving his/her wellbeing.	□	□	□	□	□
**9. They are concerned about my health and wellbeing** The healthcare and social care professionals who care for the person in my care are concerned about my health and quality of life and I feel they are committed to my wellbeing.	□	□	□	□	□
**10. They are concerned about my emotional and physical burdens** The healthcare and social care professionals who care for the person in my care are concerned about the emotional and physical burdens involved in being a caregiver and they inform me about how I can manage them.	□	□	□	□	□
**11. They inform me about health and social resources that can help me** The healthcare and social care professionals who care for the person in my care inform me about the health and social resources available (in my neighborhood, town, or city) that I can use to improve the care I provide and to take better care of myself.	□	□	□	□	□
**12. They encourage me to talk to other caregivers** The healthcare and social care professionals who care for the person in my care encourage me to participate in caregiver groups to share information and experiences on how to care for ourselves and improve our competence as caregivers.	□	□	□	□	□
**If the person in your care has been hospitalized in the last 6 months and you were already caring for them, please respond to the following statement:** **13. They care about the person in my care upon his/her arrival home after being in hospital.** After the person in my care was discharged from the hospital, they called or visited us at home to see how he/she was, what care he/she needed, and what difficulties I have faced to take care of him/her correctly.	□	□	□	□	□
**If you have needed emergency care in the last 6 months, please respond to the following statement:** **14. I know where I have to contact when the person in my care has an emergency** The professionals who care for the person in my care provided me with a telephone number I can call if complications arise in his/her disease.	□	□	□	□	□
**If the person in your care has received healthcare in his/her home in the last 6 months, please respond to the following statement:** **15. They care for the person in my care well in his/her home ** The professionals who care for the person in my care in his/her home try to solve his/her health problems in coordination with the professionals of the health center and hospital.	□	□	□	□	□
**If the person in your care has received care by the social services in the last 6 months, please respond to the following statement:** **16. Social services are coordinated with the health services to provide us with good care.** The professionals at social services who care for me talk to and coordinate with the healthcare professionals to provide us with good care.	□	□	□	□	□

In the questions below, describe how often you experience these situations (mark the appropriate box with an X). It is important to focus your answers on a specific person with whom you have had **significant experience as a caregiver in the last 6 months**.

Only respond if you have been in that situation.

Thank you for your cooperation.

### Appendix A.2. Original Spanish Questionnaire

**EXPERIENCIA DEL CUIDADOR NO PROFESIONAL DE PERSONAS CON ENFERMEDAD CRÓNICA O DEPENDENCIA**

De las preguntas que se plantean a continuación, describa la frecuencia con la que usted vive estas situaciones (marque con una X en el recuadro correspondiente). Es importante que centre sus respuestas en una persona en concreto con la que usted ha tenido una experiencia importante como cuidador/a **en los últimos 6 meses**.

	**Nunca**	**Casi Nunca**	**A Veces**		**Casi Siempre**	**Siempre**
**1. Respetan el estilo de vida de la persona que cuido** **Los profesionales sanitarios que atienden a la persona que cuido me preguntan sobre sus necesidades, costumbres y preferencias, para adaptar su plan de cuidados y tratamiento.**	□	□	□		□	□
**2. Están coordinados para ofrecernos una buena atención** **Los profesionales sanitarios que atienden a la persona que cuido en el centro de salud y los que la atienden en el hospital hablan entre ellos y se coordinan para mejorar su bienestar y su calidad de vida y los de la familia.**	□	□	□		□	□
**3. Me ayudan a informarme por Internet** **Los profesionales sanitarios que atienden a la persona que cuido me informan sobre páginas web y foros de Internet de los que me puedo fiar para conocer mejor su enfermedad, el tratamiento y las consecuencias que pueden tener en su vida.**	□	□	□		□	□
**4. Ahora sé cuidar mejor** **Con el apoyo de los profesionales sanitarios y sociales que atienden a la persona que cuido, ha mejorado mi confianza y mi capacidad para cuidarla, manejar sus problemas de salud y afrontar mejor su situación.**	□	□	□		□	□
**5. Me preguntan y me ayudan a seguir el plan de tratamiento de la persona que cuido** **Reviso con los profesionales sanitarios que atienden a la persona que cuido el cumplimiento de su plan de cuidados y tratamiento y, si tengo dudas, me las aclaran.**	□	□	□		□	□
**6. Acordamos los objetivos más importantes de sus cuidados para manejar mejor su enfermedad** **He podido comentar y pactar con los profesionales sanitarios que atienden a la persona que cuido cuáles son sus problemas de salud y sociales más importantes y cómo manejarlos adecuadamente para mantener su calidad de vida.**	□	□	□		□	□
**7. Se aseguran de que toma la medicación correctamente** **Los profesionales sanitarios que atienden a la persona que cuido me informan de cómo administrarle la medicación y revisan conmigo si la está tomando correctamente y cómo le está sentando.**	□	□	□		□	□
**8. Se preocupan por el bienestar de la persona que cuido** **Los profesionales sanitarios y sociales que atienden a la persona que cuido se preocupan por su calidad de vida y los veo comprometidos para que mejore su bienestar.**	□	□	□		□	□
**9. Se preocupan por mi salud y bienestar** **Los profesionales sanitarios y sociales que atienden a la persona que cuido se preocupan por mi salud y mi calidad de vida y los veo comprometidos con mi bienestar.**	□	□	□		□	□
**10. Se preocupan por mi sobrecarga emocional y física** **Los profesionales sanitarios y sociales que atienden a la persona que cuido se preocupan por la sobrecarga emocional y física que conlleva ser cuidador y me informan sobre cómo puedo prevenirla.**	□	□	□		□	□
**11. Me informan de recursos sanitarios y sociales que me pueden ayudar ** **Los profesionales sanitarios y sociales que atienden a la persona que cuido me informan sobre los recursos sanitarios y sociales de que dispongo (en mi barrio, ciudad o pueblo) y que me pueden ayudar a cuidarla mejor y cuidarme yo/mejor.**	□	□	□		□	□
**12. Me animan a hablar con otras personas cuidadoras** **Los profesionales sanitarios y sociales que atienden a la persona que cuido me animan a participar en grupos de personas cuidadoras para compartir información y experiencias sobre cómo cuidarnos y mejorar nuestra competencia como cuidadores.**	□	□	□		□	□
**Si la persona que usted cuida ha estado ingresada en el hospital en los últimos 6 meses y usted ya la estaba cuidando, por favor, responda a la siguiente pregunta:** **13. Se preocupan por la persona que cuido al llegar a casa tras su hospitalización.** **Después de que le dieran el alta del hospital a la persona que cuido, nos han llamado o visitado en casa para ver cómo se encontraba, qué cuidados necesitaba y con qué dificultades me he encontrado para cuidarla correctamente.**	□	□	□	□		□
**Si usted se ha encontrado necesitado de ayuda urgente en los últimos 6 meses, por favor, responda a la siguiente pregunta:** **14. Sé dónde tengo que contactar cuando la persona que cuido tiene una urgencia** **Los profesionales que atienden a la persona que cuido me han facilitado un número de teléfono dónde puedo contactar si se presentan complicaciones en su enfermedad.**	□	□	□	□		□
**Si la persona que usted cuida ha recibido atención sanitaria en su domicilio en los últimos 6 meses, por favor, responda a la siguiente pregunta:** **15. Atienden bien a la persona que cuido en su domicilio ** **Los profesionales que atienden a la persona que cuido en su casa tratan de solucionar sus problemas de salud de forma coordinada con los profesionales del centro de salud y del hospital.**	□	□	□	□		□
**Si la persona que usted cuida, ha recibido atención de los servicios sociales municipales en los últimos 6 meses, por favor, responda a la siguiente pregunta:** **16. Los servicios sociales municipales están coordinados con los servicios sanitarios para ofrecernos una buena atención** **Los profesionales que me atienden en los servicios sociales hablan y se coordinan con los profesionales sanitarios para ofrecernos una buena atención.**	□	□	□	□		□
